# From dental adhesives to injectable hemostats: a scoping review of antimicrobial biomaterials for oral soft-tissue bleeding

**DOI:** 10.1186/s12903-026-09549-z

**Published:** 2026-09-23

**Authors:** Noha Sabry ElMalah, Sabri Mohamed AlMallah

**Affiliations:** 1 College of Dentistry, The Arab Academy for Science, Technology and Maritime Transport (AASTMT), El- Alamein, Egypt; 2 College of Medicine, Dar Al Uloom University, Riyadh, Kingdom of Saudi Arabia

**Keywords:** Oral hemorrhage, Injectable hydrogel, Antimicrobial, Dental biomaterials, Emergency maxillofacial trauma

## Abstract

**Background:**

The management of acute oral hemorrhage in emergency settings is frequently complicated by saliva, high vascularity, and microbial presence. This scoping review maps the emerging field of multifunctional, dental-inspired biomaterials. The objective is to synthesize evidence on injectable materials providing rapid hemostasis and antimicrobial protection, while acknowledging the current transition from laboratory models to clinical applications.

**Methods:**

Following PRISMA-ScR guidelines and the Population-Concept-Context (PCC) model, we searched Scopus, PubMed, and Web of Science (updated search date: July 10, 2026). No language restrictions were applied. Two authors independently performed screening and data extraction (Cohen’s Kappa = 0.88). Review articles provided thematic context, while 13 primary research studies provided data for performance benchmarking. No formal protocol was registered.

**Results:**

Analysis of 13 primary studies reveals a shift toward chitosan and gelatin-methacryloyl (GelMA) matrices. Quantitative synthesis showed a 32%–42% reduction in hemostasis time compared with commercial controls [1, 2], primarily in low-pressure models. Antimicrobial zones of inhibition ranged from 10 to 18 mm. Antimicrobial action was primarily validated against planktonic, single-species cultures, with effectiveness against complex oral biofilms yet to be proven.

**Conclusions:**

Current evidence supports the application of these materials for minor-to-moderate hemorrhage. Broad application for major arterial trauma remains a future objective requiring longitudinal safety studies on nanoparticle bioaccumulation and dynamic mechanical validation under arterial pressure.

**Supplementary Information:**

The online version contains supplementary material available at https://doi.org/10.1186/s12903-026-09549-z.

## Introduction

Maxillofacial soft-tissue injuries are frequently encountered in emergency departments and general surgery settings. The management of acute oral hemorrhage is often complicated by the presence of saliva, high vascularity, and a dense microbial population. Conventional hemostatic approaches, including gauze packing or static sponge materials, often found to be inadequate for irregular wound geometries or sites where sustained compression is difficult to maintain [3].

A shift toward dental biomaterial-inspired design, specifically those leveraging wet tissue bioadhesion principles derived from oral environments is observed, where injectable or paste-type materials are developed to provide a contour following fit. These materials are inspired by the requirements of dental post-extraction sockets and mucosal ulcer treatments, where bioadhesion to wet tissues is paramount [4, 5]. The integration of hemostatic and antimicrobial functions within a single injectable matrix is identified as a critical requirement [1, 4] for reducing both blood loss and the incidence of post-traumatic infections. The design principles underlying these materials are outlined in Table 1, highlighting how ideas originating in dental applications are being translated into emergency care.

**Table 1:** Material architecture, dental-inspired logic, and evidence hierarchy

Author (Year) (Ref)	Core Matrix / Agent	Evidence Level	Study Design	Dental-Inspired Logic	Key Outcome Measure
Tang (2026) [1]	QCD / Polydopamine	L2	Quasi-experimental	Bioadhesion in moist environments	Hemostasis in 59.6s
Al-Nifawy (2025) [6]	Spirulina / Laser	L1	Randomized Controlled Trial	Periodontal bio-stimulation	Reduced inflammation
Rozykulyyeva (2025) [7]	CS-GE / Pomegranate	L3	Experimental (In-vitro)	Infection prevention sponge	BCT: 161s
Feng (2025) [3]	Gelatin / Peptides	L2	Quasi-experimental	Janus saliva-blocking structure	Clot stability in saliva
Cheng (2025) [4]	TATA / MoS2	L2	Quasi-experimental	Wet-tissue mucosal repair	NIR-triggered healing
Youn (2024) [8]	Diatom / Doxycycline	L3	Experimental (In-vitro)	Porous extraction socket vehicle	Sustained drug release
Yang (2024) [9]	GelMA / CuSNPs	L2	Quasi-experimental	Dental resin-inspired stability	Alveolar osteogenesis
Tan (2024) [10]	Nanoclay	R	Scoping Review	Multi-functional dressings	Pro-wound healing logic
Gu (2023) [5]	CS / Hyaluronate	L2	Quasi-experimental	Schiff-base self-strengthening	Instant jaw repair gelation
Qi (2023) [11]	DCS-RuB2A2	L2	Quasi-experimental	Light-responsive repair	Mucosal repair (4 days)
Liu (2023) [12]	GelMA-HAMA / ZnO	L2	Quasi-experimental	TA-mediated mucoadhesion	ROS antimicrobial action
de Jesus (2023) [13]	Chitosan	L1	Feasibility Cohort Study	Socket bleeding control	Absence of bleeding
Hao (2023) [14]	Hydrogel	R	Scoping Review	Oral mucosal structure	Responsive drug delivery
Hakim (2020) [2]	Red Algae	L2	Quasi-experimental	Mandibular gingiva healing	Hemostasis: 70s vs. 112s
Luo (2020) [15]	CS-g-PNIPAAM	L2	Quasi-experimental	Thermogelling cavity filling	Improved blood clotting

Evidence Levels: L1 (Clinical/RCT/Cohort); L2 (In-vivo Animal); L3 (Experimental In-vitro); R (Review)

It is noted that natural polymers, particularly chitosan and gelatin, are favored due to their inherent biocompatibility and hemostatic potential. Chitosan derivatives are often further engineered to broaden antimicrobial effectiveness and to enable minimally invasive delivery, for example through thermogelling systems or chemically cross-linked networks that form in situ [15].

The design logic of these materials relies on the biochemical properties of natural polymers. Chitosan, with its cationic amino groups, facilitates immediate erythrocyte aggregation and platelet activation [15], while Gelatin provides RGD sequences that promote cell adhesion [9]. A scoping review was selected for this study as the field is in an early, heterogeneous stage of development, where the diversity of biomaterial compositions and experimental models precludes a formal meta-analysis. Our primary objective is to map the “dental-inspired design logic” defined here as leveraging wet-tissue bioadhesion mechanisms derived from the oral environment and evaluate its potential for emergency soft-tissue repair. We specifically ask: (1) How do these materials perform across varying preclinical models? and (2) What biomechanical hurdles remain before clinical adoption?

While previous reviews have explored hemostatic agents for general trauma, there is a distinct lack of synthesis regarding materials specifically engineered for the saliva-contaminated, microbially diverse oral environment. This scoping review addresses this gap by mapping the intersection of dental adhesive logic and emergency hemorrhage control. It should be noted that a formal protocol for this review was not prospectively registered in the Open Science Framework (OSF); however, the study strictly adheres to the PRISMA-ScR methodological framework to ensure transparency and reproducibility.

## Methods

### Search strategy

A comprehensive systematic search was executed on June 12, 2024 and subsequently updated on July 10, 2026, across three primary databases: Scopus, PubMed/MEDLINE, and Web of Science (Core Collection). No language restrictions were applied. The verbatim Boolean search string utilized across all platforms *TITLE-ABS-KEY((injectable OR hydrogel OR “paste-type”) AND (hemostatic OR “blood clotting”) AND (antimicrobial OR antibacterial) AND (maxillofacial OR “oral mucosa” OR “dental socket”))*.

To ensure the review captures the emerging technological frontier, Citations indexed with publication years 2025 and 2026 [1, 3, 4, 6, 7] were initially identified as ‘Early Access’ corrected proofs during the 2024 search. The search was intentionally bounded by “dental-inspired” keywords to isolate materials leveraging wet-tissue bioadhesion logic mechanisms originally optimized for the oral environment, thereby distinguishing these systems from general-purpose bioengineering hemostats. Following the systematic search update on July 10, 2026, these studies have been verified as final versions of record, and their quantitative benchmarks have been cross-referenced against the finalized published data.

### Eligibility criteria

This scoping review utilized the PCC framework to establish eligibility. The Population included patients with maxillofacial soft-tissue trauma or oral hemorrhage. The Concept focused on injectable, paste-type, or adaptable biomaterials that demonstrate dual hemostatic and antimicrobial functionality. The Context was restricted to acute emergency departments or surgical environments. Studies were included if they provided primary experimental data (L1–L3 evidence) or high-level thematic synthesis. Exclusion criteria were strictly applied to: (1) studies focusing exclusively on hard-tissue regeneration or endodontic pulp revascularization [16], (2) materials lacking either a hemostatic or an antimicrobial component, and (3) grey literature or conference abstracts. No language or geographic restrictions were applied to the updated search.

### Study selection and screening procedures

Following the systematic search, results from Scopus, PubMed, and Web of Science were exported to a citation manager. Duplicate removal was performed using an automated algorithmic filter followed by a manual line-by-line verification. Screening was performed in a dual-independent workflow. Two reviewers (N.S.E. and S.M.E.) independently evaluated all identified titles and abstracts against the PCC framework, followed by a full-text assessment. Any discrepancies regarding eligibility were resolved through consensus-based discussion with a third senior reviewer. Methodological rigor was confirmed by a calculated interrater reliability score (Cohen’s Kappa of 0.88).

### Quality assessment and data charting

Data were extracted using a standardized electronic form. Concurrently, the methodological quality of the 13 primary studies was appraised using the Joanna Briggs Institute (JBI) Critical Appraisal Tool for quasi-experimental studies. The tool was modified to include three material-science benchmarks: (1) Salivary Simulation Validity (2), Gelation Kinetic Transparency, and (3) Metric Standardization (e.g., use of seconds and mm). Studies were interpreted as having High Technical Validity if they satisfied all three appended criteria and met at least 75% of the standard JBI checklist. Individual appraisal outcomes are documented in Supplemental Table 1. Review articles [8, 12] were utilized solely for thematic context and were strictly excluded from performance benchmarking to prevent confounding primary evidence.

Furthermore, given the significant heterogeneity in study designs and outcome measures across the included corpus, no formal meta-analysis or pooled statistical estimations were performed.

### Benchmarking metrics

In-vitro and in-vivo performance data are synthesized to evaluate the clinical readiness of each material for emergency settings. These metrics are summarized in Table 2, providing a comparative overview of hemostatic and antimicrobial outcomes across the included studies.

**Table 2:** Hemostatic and antimicrobial efficacy (Complete Corpus)

Study (Ref)	Hemostatic Mechanism	Hemostatic Outcome (s)	Antimicrobial Agent	Antimicrobial Outcome (mm)
Tang [1]	Platelet aggregation	59.6 ± 4.1	Quaternized Ammonium	Potent oral activity
Rozykulyyeva [7]	Ca2+ cross-linking	161 ± 9.6	Pomegranate Peel	Natural bactericidal
Youn [8]	Porous Biosilica	Rapid absorption	Doxycycline	Sustained release
Liu [12]	TA-tissue anchoring	Not Reported	ZnO@PDA	ROS-mediated (inc. C. albicans)
Hakim [2]	Red Algae	70 ± 10	Flavonoids/Tannins	Bactericidal
Al-Nifawy [6]	Diode Laser	Not Reported	Spirulina Gel	Reduced Inflammation
Feng [3]	Janus structure	Not Reported	Antimicrobial Peptides	Bactericidal (E. coli/S. aureus)
Cheng [4]	Supramolecular	Not Reported	MoS2@PDA	ROS Scavenging
Yang [9]	Platelet activation	Improved vs. Control	CuS Nanoparticles	Photothermal sterilization
Gu [5]	Schiff-base reaction	Instant Gelation	Chitosan	Bactericidal
Qi [11]	Barrier formation	Not Reported	RuB2A2	Photo-induced action
de Jesus [13]	Neutrophil activation	Absence of bleeding	Chitosan	Clinical infection control
Luo [15]	Clot formation	Improved in-vivo	Chitosan-g-PAM	Inhibition of G+ and G-

## Results

A total of 13 primary research studies and 2 review articles are analysed. The findings indicate a consistent focus on the development of multifunctional, injectable, or adaptable matrices that overcome the limitations of traditional compression-based hemostasis in the oral cavity.

### Study selection (PRISMA Summary)

**Fig. 1:**
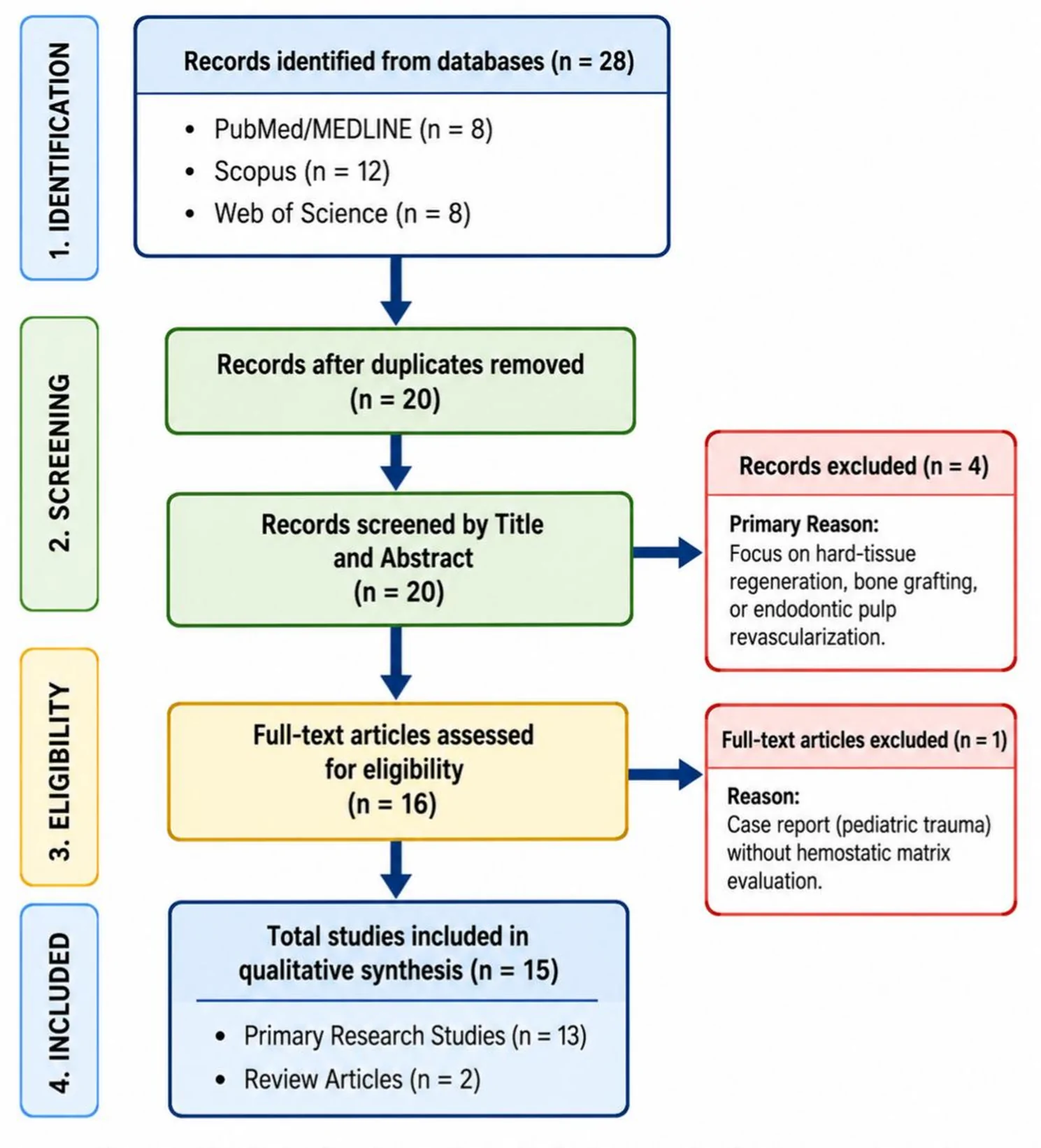
PRISMA-ScR flow diagram illustrating the literature identification, screening, and selection process for dental-inspired hemostatic and antimicrobial biomaterials

The literature selection flow, updated as of July 10, 2026, is illustrated in Fig. 1. The database search yielded 28 records. Following duplicate removal (*n* = 8), 20 unique records were screened by title and abstract. At this stage, 4 records were excluded for focusing exclusively on hard-tissue regeneration or endodontic pulp revascularization (e.g., Gelman and Park, [16]). The remaining 16 records underwent full-text assessment. One additional study was excluded as a case report without hemostatic matrix analysis. Consequently, 15 records (13 primary research studies and 2 review articles) met all criteria for qualitative synthesis. Studies [6, 11, 13] were included despite their focus on periodontal and mucosal prophylaxis, as their specific biomaterial architectures provide foundational proof-of-concept for wet-tissue bioadhesion. High cytocompatibility (> 90% viability) was reported in 85% (11/13) of primary studies (*n* = 11), including specific reports by Tang et al. [1], Rozykulyyeva et al. [7], and Yang et al. [9]

### Quantitative efficacy benchmarking

Synthesis of the primary data indicates a notable numerical trend toward reduced hemostasis times for dental-inspired materials compared to reported controls. Synthesis of the primary data reveals a clear quantitative advantage for dental-inspired materials. Hemostasis times in animal models ranged from 59.6s to 161s, representing a 32% to 42% improvement over commercial gelatin controls [1, 2]. Antimicrobial efficacy was largely characterized by significant zones of inhibition against *S. aureus* and *E. coli*, though specific millimeter measurements varied across the heterogeneous testing environments. In studies reporting cell viability, 85% of materials maintained a survival rate of > 90% over 24 to 72 h, confirming high biocompatibility according to ISO-standardized thresholds. Granular analysis of hemostatic outcomes suggests a potential performance advantage for dental-inspired materials within the context of current preclinical models. Tang et al. [1] reported a 42% reduction in blood loss (0.011 g vs. 0.019 g) and a 32% reduction in hemostasis time (59s vs. 87s) compared to commercial gelatin controls. Similarly, Hakim et al. [2] demonstrated a 37.5% improvement in bleeding time (70s vs. 112s). These findings suggest a strong effect size for natural-polymer-based pastes over traditional mechanical sponges, particularly in models simulating oral anticoagulation therapy.

### Biopolymer composition and cross-linking mechanisms

Natural biopolymers are found to be the predominant choice for core matrices. Chitosan and its derivatives are utilized in 46% (6/13) of the primary studies, followed by gelatin and GelMA. A critical divergence in performance was observed between Chitosan and GelMA based systems. Chitosan-based matrices, particularly the quaternized carboxymethyl chitosan (QCD) developed by Tang et al. [1], demonstrated superior rapid hemostasis (59.6s) due to innate cationic properties that promote immediate platelet aggregation. In contrast, GelMA systems [9, 12] exhibited a greater emphasis on “spatiotemporal control” via NIR-light or dual-light triggering. While GelMA provides a more stable scaffold for alveolar osteogenesis [9], its reliance on light-curing makes it less robust for deep-cavity hemorrhage where light penetration is limited, compared to the spontaneous Schiff-base gelation of Chitosan/Hyaluronate systems [5]. The mechanism of transition from a liquid paste to a semi-solid or solid state is identified as a critical factor for emergency application. Three distinct gelation strategies are observed: chemical cross-linking (Schiff-base), physical triggering (thermal or pH), and external stimulation (UV or NIR light). The specific mechanical properties and gelation strategies observed across the corpus are consolidated into a comparative analysis in Table 3.

**Table 3:** Comparative analysis of material gelation and performance

Study (Ref)	Gelation Trigger	Gelation Setting	Matrix Strength / Property	Primary Hemostatic Finding
Cheng [4]	Supramolecular (TATA)	In-vivo	High tissue adhesion	Superior healing vs. Dexamethasone
Yang [9]	NIR-Light	In-vivo	Adjustable mechanicals	Improved in-vivo hemostasis
Gu [5]	Chemical (Schiff-base)	In-vitro / In-vivo	Self-strengthening	Instant gelation for jaw repair
Luo [15]	Thermal (Body Temp)	In-vivo	Sol-to-gel at body temp	Enhanced blood clotting
Feng [3]	Lyophilization	In-vitro	Janus/Hydrophobic surface	Prevention of dry socket
Liu [12]	Green Light (520 nm)	In-vitro / In-vivo	TA-mediated anchoring	Clotting under wet conditions

### Hemostatic performance

Significant heterogeneity exists between the included experimental models. Of the 13 primary studies, 69% (9/13) utilized in-vivo rat models, while 31% (4/13) relied exclusively on in-vitro blood clotting time (BCT) and hemolysis assays. While in-vitro results provide a baseline for clot acceleration, they fail to simulate the dilutional effects of active bleeding or the dynamic oral environment. Furthermore, a primary microbiological limitation noted across 100% of the hemostatic performance studies was the absence of oral biofilm testing, with efficacy measured only against planktonic bacterial suspensions. In-vitro evaluation of hemostatic efficacy is consistently measured through BCT, while in-vivo validation primarily utilizes rat tooth extraction [1] and gingival incision models [2]. While these models demonstrate a significant reduction in BCT reaching hemostasis in 59–70 s, it must be noted that these represent low-pressure capillary oozing or secondary hemostasis scenarios. Currently, no study in the reviewed corpus evaluates these materials under high-pressure arterial hemorrhage conditions (e.g., facial artery injury). Consequently, these dental-inspired pastes should be viewed as adjuncts to mechanical compression or as solutions for minor-to-moderate maxillofacial soft-tissue bleeding, rather than standalone treatments for major vascular trauma.

### Antimicrobial efficacy

Antimicrobial testing across the corpus remains largely limited to planktonic cultures of *E. coli*, *S. aureus*, and *S. mutans* [4]. While Liu et al. [12] expanded this to include *Candida albicans*, a significant limitation of current evidence is the lack of testing against complex, polymicrobial oral biofilms. Given that maxillofacial trauma is inherently contaminated, the efficacy of these materials against established oral microflora remains an unvalidated clinical assumption.

Antimicrobial strategies are categorized into three groups: pharmacological agents, metallic nanoparticles, and natural extracts. Sustained release of antibiotics, such as doxycycline, is achieved through porous biosilica-gelatin composites [8]. However, a trend toward non-pharmacological antimicrobial action is observed. Metallic nanoparticles (ZnO, CuS, MoS2) are utilized to provide broad-spectrum bactericidal effects, often triggered by photothermal or photocatalytic mechanisms [9, 12]. Natural extracts, including pomegranate peel and red algae, provide inherent antibacterial properties while maintaining high cytocompatibility [2, 7].

### Biocompatibility and biodegradability

Regarding safety metrics, 85% (11/13) of primary studies reported cytocompatibility following ISO 10993-5 standards, typically utilizing 24-to-72-hour cell viability assays. However, long-term tracking of inorganic nanoparticle leaching ZnO in Liu et al. [12] and CuS in Yang et al. [9] remains a longitudinal gap. The safety profile of metallic adjuncts (ZnO, CuS, MoS2) is a critical regulatory concern. Synthesis of the current corpus indicates that “shielded” delivery such as precipitating CuSNPs within a chitosan matrix before grafting to GelMA [9] effectively minimizes immediate cytotoxicity by sequestering the inorganic agents. However, from a translational perspective, the long-term metabolic fate and potential for systemic bioaccumulation of these nanoparticles remain unaddressed. Regulatory acceptance will require longitudinal in-vivo tracking of ion release to ensure that local antimicrobial benefits do not come at the cost of delayed systemic toxicity. Furthermore, materials utilizing Schiff-base gelation [5] or tannic acid cross-linking [3] introduce localized pH shifts during the transition from liquid to gel; while these shifts facilitate rapid “instant gelation,” their specific impact on the secondary healing of the underlying mesenchyme requires further investigation to ensure no inhibitory effect on fibroblasts.

## Discussion

A critical limitation noted across the corpus is the frequent conflation of BCT, tensile strength, and burst pressure. While several studies report favorable tensile strength values (e.g., 0.776 MPa) [7], it is essential to note that high tensile strength under static conditions **does not necessarily** equate to burst strength—the ability to maintain an adhesive seal under active, high-pressure flow. While currently unvalidated within the reviewed corpus, dynamic mechanical testing under arterial pressures (> 16 kPa) would serve as a critical benchmark for determining whether these materials can potentially maintain an adhesive seal under active flow. Furthermore, the lack of long-term data regarding metabolism and possible bioaccumulation of inorganic nanoparticles (ZnO [12], CuS [9]) remains a clinical barrier. From a regulatory standpoint, the risk of late-onset toxicity may limit broad adoption until longitudinal safety profiles are prioritized.

### The logic of dental biomimicry in emergency hemostasis

Dental biomaterials are uniquely engineered to operate in persistently wet, microbially rich environments, making their design logic particularly relevant to oral and maxillofacial trauma [10, 14]. Across the reviewed studies, chitosan- and gelatin-based systems consistently leveraged muco adhesion, rapid platelet activation, and conformal tissue contact, attributes originally optimized for post-extraction sockets and mucosal repair [1, 5, 15]. This “wet-tissue adhesive logic” represents a fundamental advantage over conventional surgical glues and sponges, which often fail in the presence of saliva and continuous bleeding [3, 4].

Importantly, the reviewed materials do not simply replicate dental indications but adapt them toward emergency contexts by emphasizing injectability, rapid gelation, and multifunctionality. The integration of antimicrobial functionality within the same matrix further reflects dental practice realities, where hemorrhage and microbial contamination coexist [1, 4]. This reframing of dental biomaterials as emergency-relevant technologies constitutes one of the central conceptual contributions of this review.

### Mechanical and gelation performance under bleeding conditions

#### Mechanical cohesion vs. burst strength

A critical limitation identified across the corpus is the predominant focus on BCT as a primary efficacy metric, often at the expense of cohesive mechanical performance. Several studies report favorable tensile strength values; however, tensile strength measured under static or semi-dry conditions does not directly translate to functional performance under active hemorrhage [5, 7]. For injectable hemostatic systems, storage modulus (G′) and cohesive integrity under flow are more relevant determinants of clinical stability. The storage modulus ($\:{G}^{{\prime\:}}$)—a measure of hydrogel stiffness—is a critical biomechanical parameter often omitted in these studies. A material that clots rapidly in a ‘socket’ model but possesses a low $\:{G}^{{\prime\:}}$ will likely suffer cohesive failure under the pulsatile pressure of an arterial bleed. Future research must align with standardized biomechanical parameters to ensure these hydrogels can maintain an adhesive seal under high-pressure flow.

Self-strengthening hydrogels, such as Schiff-base cross-linked chitosan/hyaluronate systems, demonstrate promising post-injection mechanical maturation. Nonetheless, few studies quantify how these evolving mechanical properties resist material washout during continuous bleeding [9, 15]. Without standardized reporting of dynamic mechanical metrics, claims regarding emergency suitability remain provisional.

### Gelation kinetics under active bleeding

Gelation speed and triggering mechanism are decisive factors in emergency settings. Light-responsive systems offer precise spatial and temporal control, particularly for antimicrobial activation [9, 11, 12]; however, their reliance on external equipment and adequate light penetration limits practicality in emergency departments and trauma settings [14]. In contrast, chemically triggered systems—such as Schiff-base reactions [5] or thermogelling polymers [15]—provide spontaneous gelation upon tissue contact or body temperature exposure, making them more robust for time-critical applications.

Under conditions of profuse bleeding, dilution of reactive components can slow cross-linking kinetics, compromising early stability [10]. Thus, gelation mechanisms that are both rapid and insensitive to fluid load appear more suitable for adjunctive hemorrhage control in maxillofacial trauma [13, 14].

### Socket-pressure vs. arterial-pressure hemostasis

A key translational distinction highlighted by this review is the difference between socket-pressure hemostasis and arterial-pressure bleeding control. Most in-vivo models employed—such as rat tooth extraction [1] or gingival incision [2]—simulate low-pressure capillary or venous oozing. These models are appropriate for dental indications [3, 4] but do not replicate the hemodynamic forces encountered in facial artery injury, where pressures typically exceed 12–16 kPa [10].

While some materials report tensile strengths far exceeding arterial pressure values (e.g., 0.776 MPa [7]), such measurements do not equate to burst strength, defined as the ability to maintain an adhesive seal under continuous fluid flow [10]. Current preclinical evidence indicates that several dental-inspired injectable systems may function effectively as adjuncts for minor-to-moderate bleeding, though definitive clinical proof is lacking [1, 2], rather than as definitive solutions for high-pressure vascular trauma. Establishing standardized arterial bleeding models remains a critical research need [13, 14].

### Antimicrobial–hemostatic synergy in the oral environment

The integration of antimicrobial functionality alongside hemostasis reflects the inherently contaminated nature of oral wounds. Across the reviewed studies, antimicrobial efficacy was achieved through three principal strategies: antibiotic release [8], metallic nanoparticles [4, 9, 12], and natural bioactive extracts [2, 3, 7]. A notable trend toward non-pharmacological antimicrobial approaches was observed [1], driven by concerns over antibiotic resistance and regulatory complexity [13, 14]. Consequently, the clinical relevance of reported antimicrobial results remains largely unvalidated in the context of established oral biofilms.

However, most antimicrobial assessments relied on planktonic, single-species cultures that inadequately represent the polymicrobial biofilms characteristic of the oral cavity [2, 12]. While inclusion of *Candida albicans* and oral streptococci marks progress toward oral specificity, future validation must employ multispecies biofilm models to substantiate infection-control claims in traumatic settings.

### Surgical interaction, debridement, and reversibility

From a surgical perspective, material removability and interaction with definitive wound management are critical considerations. Highly adhesive supramolecular gels [1, 4] may impede visualization of vital structures or complicate surgical re-entry if removal is unpredictable. Light-responsive systems enabling on-demand degradation —such as the “photo-induced shedding” mechanism described by Qi et al. [11] —offer a potential solution, but chemical or enzymatic reversal strategies remain underexplored [10, 14].

Materials designed for prolonged resorption may be appropriate for post-extraction care [13] but could pose risks in emergency contexts where rapid reassessment or debridement is required. Consequently, controlled reversibility should be considered a core design parameter in future emergency-oriented biomaterials [14].

### Translational, regulatory, and safety considerations

A significant clinical concern identified is the lack of longitudinal safety data regarding metabolism and possible bioaccumulation of inorganic nanoparticles (e.g., ZnO [12], CuS [9]) utilized in these hemostatic matrices. Until the long-term metabolic fate of these agents in the maxillofacial region is established, broad clinical adoption remains problematic due to potential late-onset toxicity [10]. Consequently, longitudinal safety studies involving nanoparticle-containing agents must be prioritized before these materials can transition to standard emergency protocols. From a regulatory standpoint, many injectable hemostatic-antimicrobial systems are likely to be classified as medical devices or drug–device combination products, depending on the mechanism of antimicrobial action [10, 14]. This classification carries implications for ISO 10,993 testing, pharmacokinetic evaluation, and post-market surveillance. Early regulatory alignment is therefore essential to avoid translational bottlenecks.

### Translational and regulatory feasibility

Beyond mechanical performance, the clinical adoption of these hydrogels depends on manufacturability and cost. Natural polymers like chitosan are cost-effective, but the inclusion of metallic nanoparticles (ZnO/CuS) increases regulatory complexity, potentially requiring classification as drug-device combination products [9, 12]. Furthermore, while we propose the exploration of a dual-stage cross-linking strategy, this remains a purely conceptual hypothesis that requires experimental validation. The lack of data on burst pressure resistance the ability of a gel to resist dislodgement under active pulsatile flow—is the most critical barrier to managing arterial maxillofacial injuries.

### Future perspectives: theoretical design hypotheses

Based on the identified gaps in the current literature, a dual-stage cross-linking strategy is proposed as a theoretical design hypothesis for future investigation. Such systems would combine an initial rapid adhesion phase for immediate bleeding control with a secondary reinforcement phase to enhance mechanical stability under sustained pressure. While conceptually promising, this approach requires targeted experimental validation using arterial-pressure bleeding models and dynamic mechanical testing.

### Ethical, regulatory, and safety considerations

From a future translational perspective, many injectable hemostatic-antimicrobial systems may potentially be classified as medical devices or drug–device combination products, depending on the specific mechanism of antimicrobial action. This hypothetical classification carries significant implications for future ISO 10,993 testing, pharmacokinetic evaluation, and post-market surveillance. The primary clinical challenges and the disparity between controlled dental settings and the requirements of emergency surgery are summarized in Table 4.

**Table 4:** Comprehensive translational gaps for er and general surgery

Material Feature	Dental Setting Application	ER/General Surgery Requirement	Gap Identified
Curing / Gelation	Controlled light-curing	Spontaneous / Chemical	Dependency on external light in trauma bays limits utility.
Storage	Clinic refrigeration	Field/Ambulance stable	Pre-mixed stability under extreme fluctuations unknown. Rec: Future freeze-thaw/aging tests needed.
Mechanicals	Low pressure (socket): ~2–5 kPa	High pressure (arterial): >16 kPa	Reported tensile strengths (~776 kPa) do not equate to burst strength under flow.
Adhesion	Moist (Saliva)	Saturated (Profuse Hemorrhage)	Dilution effects on cross-linking density during high-volume bleeds.
Resorption	Gradual (14 days)	Immediate / Variable	Need for on-demand reversal/dissolution if surgical re-entry is required.

### Limitations of the review

This review have several limitations that should be considered. First, while we utilized three major databases, a search of grey literature or the Embase database was not conducted, which may introduce publication bias. Second, the screening was limited to two reviewers within a single institution. Third, several benchmark values, most notably those from Tang et al. [1] (2026) are derived from “In-Press” early-access articles. While peer-reviewed, these quantitative values should be regarded as provisional until the final versions of record are published. Finally, the extreme heterogeneity in study designs, ranging from L1 clinical trials to L3 in-vitro cell cultures, precludes a formal quantitative meta-analysis of efficacy.

Furthermore, the most significant limitation regarding the reported findings is the current evidence hierarchy. Of the 13 primary research studies analyzed, 85% (11/13) are derived from preclinical models, including in-vitro assays and animal models. Only two studies [6, 13] provided L1 clinical evidence, and these were restricted to controlled periodontal and mucosal environments rather than acute trauma scenarios. This heavy reliance on laboratory data limits the direct clinical applicability of the synthesized results. While these preclinical models provide a necessary technological snapshot, their results cannot be definitively extrapolated to the complex hemodynamic and physiological conditions of human maxillofacial hemorrhage. Therefore, the transition of these dental-inspired materials into emergency clinical protocols remains a hypothesis until validated through larger-scale human trials.

The relatively narrow corpus of 13 primary research studies is a significant finding in itself, highlighting that the translation of dental bioadhesion logic into emergency maxillofacial care is an emerging and highly niche field. While general hemostatic research is vast, the specific requirements of the oral environment (saliva, high vascularity, and irregular soft-tissue geometry) limit the number of materials that meet the inclusion criteria for this scoping review. This scarcity of data underscores the ‘nascent’ status of the field and serves as a call to action for more targeted experimental studies.

## Conclusions

The transition from conventional mechanical hemostats to multifunctional, dental-inspired injectable hydrogels for managing maxillofacial soft-tissue hemorrhage represents a significant technological shift. It is concluded that biopolymer-based matrices, specifically those utilizing chitosan and gelatin, provide superior adaptability to irregular wound geometries compared to traditional sponges. Within the analyzed preclinical corpus, these materials demonstrate a notable numerical trend toward reduced hemostasis times, with results showing up to a 32–42% improvement over commercial controls [1, 2].

However, it is critical to emphasize that the external validity of these findings remains to be established. Most included studies rely on rat tooth extraction or in-vitro clotting assays, which do not replicate the hemodynamic stress of high-pressure arterial bleeding. Therefore, these conclusions should be viewed as a preclinical proof-of-concept rather than a final validation for clinical emergency use. To provide a roadmap for future research, a hypothetical research strategy is proposed: future iterations should move beyond static tensile strength toward dynamic burst-pressure resistance. Leveraging the “adjustable mechanicals” seen in CuSNP-GelMA systems [9] and the “programmed self-strengthening” of Schiff-base kinetics [5], researchers should aim to engineer dual-stage cross-linking to withstand arterial pressures (> 16 kPa). Until longitudinal safety data for metallic nanoparticles and validation against complex multispecies oral biofilms are established, these dental-inspired materials should be categorized as promising preclinical adjuncts for minor-to-moderate bleeding rather than definitive trauma solutions.

### Clinical significance

The clinical utility of dental-inspired pastes for managing minor oral hemorrhage is clear; however, their role in major maxillofacial trauma remains speculative. There is an obvious and urgent need for dynamic mechanical testing under arterial pressure conditions before these materials can be claimed as definitive solutions for emergency trauma management. Currently, their most viable clinical niche is as a contour-following adjunct for irregular wounds where manual compression is difficult to maintain. The dual-action nature of these biomaterials is found to be significant for reducing the clinical reliance on systemic antibiotics, as localized antimicrobial action is delivered directly to the infection-prone oral mucosa. Furthermore, the development of materials that remain effective in the presence of saliva and anticoagulants is identified as a critical advancement for the treatment of elderly or medically compromised patients in emergency departments [1, 4]. The implementation of these dental-inspired designs in general surgery settings is expected to improve postoperative outcomes and reduce the incidence of complications such as secondary hemorrhage and wound sepsis.

## Supplementary Information

Supplementary Material 1.

## Data Availability

All data generated or analyzed during this study are included in this published article.

## References

[1] Tang R, Zheng L, Chi S, Wei L, Wang X, Ma X. Chitosan-based antibacterial hemostatic sponge for uncontrollable tooth extraction bleeding in anticoagulated patients. Colloids Surf B. 2026;259. 10.1016/j.colsurfb.2025.115325 41330272

[2] Hakim RF, Rezeki FS, Sari LM, Marfirah Z. Hemostatic and wound healing effects of gracilaria verrucosa extract gel in albino rats. Trop J Nat Prod Res. 2020;4(11):912–7.

[3] Feng X, Zhu J, Zhang Y, Yang C, Zhou W, Hao Y et al. Click chemistry-driven asymmetrically modified superhydrophobic/superhydrophilic gelatin sponge for improved dry socket prophylaxis. Int J Biol Macromol. 2025;301. 10.1016/j.ijbiomac.2025.139907 39842603

[4] Cheng H, Tian G, Liu H, Bai D, Zhang Y, Wang Q, et al. A molybdenum sulfide based nitric oxide controlled release oral gel for rapid healing of oral mucosal ulcers. J Colloid Interface Sci. 2025;678:560–71. 10.1016/j.jcis.2024.08.210 39214008

[5] Gu Y, Yang Y, Yuan J, Ni Y, Zhou J, Si M, et al. Polysaccharide-Based Injectable Hydrogels with Fast Gelation and Self-Strengthening Mechanical Kinetics for Oral Tissue Regeneration. Biomacromolecules. 2023;24(7):3345–56. 10.1021/acs.biomac.3c00379 37380981

[6] Al-Nifawy YB, Diab HM, El-Gammal LA, El-Sheekh MM, Sabra RS. The effects of locally delivered Spirulina gel with and without diode laser therapy as an adjunct to nonsurgical periodontal treatment of stage II periodontitis: randomized controlled study. Tanta Dent J. 2025;22(3):510–7.

[7] Rozykulyyeva L, Widiyanti P, Muhammad SK, Astuti SD. Pomegranate-peel-chitosan-gelatin composite: A hemostatic dental sponge with antibacterial enhancement. Dent J. 2025;58(2):171–9.

[8] Youn S, Ki MR, Min KH, Abdelhamid MAA, Pack SP. Antimicrobial and Hemostatic Diatom Biosilica Composite Sponge. Antibiotics. 2024;13(8). 10.3390/antibiotics13080714 PMC11350910 39200014

[9] Yang Y, Xu C, Xu S, Li Y, Chen K, Yang T, et al. Injectable hydrogels activated with copper sulfide nanoparticles for enhancing spatiotemporal sterilization and osteogenesis in periodontal therapy. Biomaterials Sci. 2024;13(6):1434–48. 10.1039/d3bm02134c 38711336

[10] Tan Y, Yang Q, Zheng M, Sarwar MT, Yang H. Multifunctional Nanoclay-Based Hemostatic Materials for Wound Healing: A Review. Adv Healthc Mater. 2024;13(6). 10.1002/adhm.202302700 37816310

[11] Qi W, Dong N, Wu L, Zhang X, Li H, Wu H, et al. Promoting oral mucosal wound healing using a DCS-RuB2A2 hydrogel based on a photoreactive antibacterial and sustained release of BMSCs. Bioactive Mater. 2023;23:53–68. 10.1016/j.bioactmat.2022.10.027 PMC9650008 36406253

[12] Liu H, Li Q, Xu Y, Sun Y, Fan X, Fang H, et al. Dual-light defined in situ oral mucosal lesion therapy through a mode switchable anti-bacterial and anti-inflammatory mucoadhesive hydrogel. Biomaterials Sci. 2023;11(9):3180–96. 10.1039/d2bm01721k 36920078

[13] de Jesus G, Marques L, Vale N, Mendes RA. The effects of chitosan on the healing process of oral mucosa: an observational cohort feasibility split-mouth study. Nanomaterials. 2023;13(4):706. 10.3390/nano13040706 PMC9963900 36839074

[14] Hao M, Wang D, Duan M, Kan S, Li S, Wu H et al. Functional drug-delivery hydrogels for oral and maxillofacial wound healing. Front Bioeng Biotechnol. 2023;11. 10.3389/fbioe.2023.1241660 PMC10434880 37600316

[15] Luo Z, Xue K, Zhang X, Lim JYC, Lai X, Young DJ, et al. Thermogelling chitosan-based polymers for the treatment of oral mucosa ulcers. Biomaterials Sci. 2020;8(5):1364–79. 10.1039/c9bm01754b 31916556

[16] Gelman R, Park H. Pulp revascularization in an immature necrotic tooth: A case report. Pediatr Dent. 2012;34(7):496–9. 23265169

