# Supplementary material for: From dental adhesives to injectable hemostats: a scoping review of antimicrobial biomaterials for oral soft-tissue bleeding
Source: BMC Oral Health. 2026 Sep 23;26:1802. doi: 10.1186/s12903-026-09549-z (PMC13602568; doi:10.1186/s12903-026-09549-z)
Supplement: Supplementary file 1 [file 12903_2026_9549_MOESM1_ESM.docx]

**Supplemental Table 1: Technical Benchmarking and Standard Adherence**

| Study (Ref) | Core Matrix | Biocompatibility Standard | Mechanical Metric | Microbiological Strains |
| --- | --- | --- | --- | --- |
| Tang (1) | QCD | Hemolysis <5% (standard not specified) | Rapid blood absorption | *S. aureus, E. coli* |
| Rozykulyyeva (9) | CS-GE | ISO 10993-5 (In-vitro) | Tensile: 0.776 MPa | *S. aureus, E. coli* |
| Al-Nifawy (8) | Spirulina | Clinical Trial | Clinical Adhesion | Immunologic (IL-1β) |
| Liu (14) | GelMA-HAMA | In-vivo | Mucosal anchoring | *C. albicans*; *S. aureus* |
| Cheng(4) | TATA | In-vivo | High tissue adhesion | *S. mutans* |
| Gu (5) | CS/HA | In-vitro / In-vivo | Self-strengthening | *S. aureus, E. coli* |
